# The Protective Effect of Sheep Placental Extract on Concanavalin A-induced Liver Injury in Mice

**DOI:** 10.3390/molecules24010028

**Published:** 2018-12-21

**Authors:** Jingwen Liu, Suting Luo, Jun Yang, Fazheng Ren, Yu Zhao, Hailing Luo, Keshan Ge, Hao Zhang

**Affiliations:** 1 Beijing Advanced Innovation Center for Food Nutrition and Human Health, College of Food Science and Nutritional Engineering, China Agricultural University, No. 17 Qinghua East Road, Haidian, Beijing 100083, China; jingwen_liu@foxmail.com (J.L.); lstncu@163.com (S.L.); renfazheng@cau.edu.cn (F.R.); gkeshan@163.com (K.G.); 2 Beijing Laboratory of Food Quality and Safety, China Agricultural University, No. 17 Qinghua East Road, Haidian, Beijing 100083, China; 3 Caoyuanxinhe Food Company Limited, Linlang Road, Linhe, Bayannaoer 015000, Inner Mongolia, China; fdl_angel@163.com; 4 Department of Food Science, Pennsylvania State University, University Park, PA 16802, USA; zhaoyu539@gmail.com; 5 State Key Laboratory of Animal Nutrition, College of Animal Science and Technology, China Agricultural University, No. 2 Yuanmingyuan West Road, Haidian, Beijing 100193, China; luohailing@cau.edu.cn

**Keywords:** sheep placental extract, concanavalin A, immunological liver injury, serum aminotransaminase activity, pro-inflammatory cytokines, liver antioxidant capacity

## Abstract

Though the biological effects of human placental extract have been widely studied, it has limited availability and its use poses ethical problems. Thus, domestic animal placental extracts are suggested as alternatives. In this study, the protective effect of sheep placental extract (SPE) on concanavalin A (Con A)-induced liver injury was investigated. BALB/c mice were randomly divided into six groups, including one normal group and five experimental groups, which received different oral doses of SPE (0, 5, 10 and 50 mg/kg) or a mixture of amino acids for 3 days before Con A injection. Compared with Con A-induced model group, the SPE administration significantly decreased serum aminotransaminase activity, alleviated pathological changes, recovered liver antioxidant capacity and prevented the increase of nitric oxide. Secretion of pro-inflammatory cytokines in serum decreased and mRNA expression of hepatic intercellular adhesion molecule-1, interferon-inducible chemokine 10 and inducible nitric oxide synthase were downregulated, while B-cell lymphoma-2 expression increased. The administration of amino acids mixture had no significant effect in most measurements compared with the model group, which indicated proteins and peptides, rather than individual amino acid, were largely responsible for the bioactivity of SPE. The results indicate SPE has potential therapeutic effects against immune-mediated hepatitis.

## 1. Introduction

The placenta is an important mammalian organ during pregnancy, functioning as a provider of hormones, nutrients and oxygen to the fetus. It also can modulate maternal immune responses and contain various biologically active components, such as a variety of regulatory peptides, hormones, growth factors and cytokines [1]. Placental extract has been widely used in traditional medicine for wound healing and as an anti-inflammatory agent [2,3]. Moreover, the human placental extract has been used to treat liver disease for more than 40 years [4]. It can stimulate liver regeneration and its main active component is hepatocyte growth factor [5]. Jung et al. demonstrated human placental extract exhibited therapeutic effects in carbon tetrachloride-induced liver damage through reducing fibrosis and regulating inflammation [6]. Apart from traditional medicine, human placenta can be applied in functional foods as well as cosmetics. However, the limited availability and ethical problems prevent its wide application.

Some reports have demonstrated animal placental extracts exerted similar functions as human placenta. Park et al. reported that porcine placental extract had significant immunomodulatory effects both in vitro and in vivo [7]. In addition, sheep placental extract could inhibit lymphocyte activation and the proliferative response to lectins [8]. Thus, placenta from domestic animals such as sheep, pigs and cows can be chosen as alternatives to replace human placenta. Sheep placenta is widely available in China due to abundant resources of sheep farming. Considering the immunoregulatory and liver protective activities of placental extracts, it is plausible that sheep placental extract (SPE) may protect liver from concanavalin A (Con A)-induced injury. Con A is a lectin derived from jack beans and Con A-induced liver injury can serve as a model for immunologically induced hepatocyte injury, which is characterized by high serum levels of aminotransferase and hepatic pathological changes [9].

In this study, we investigated the protective effects of SPE on Con A-induced liver injury in vivo. Biochemical markers and cytokine contents in serum, such as alanine transaminase (ALT), aspartate transaminase (AST), tumour necrosis factor-α (TNF-α), interferon-γ (IFN-γ), interleukin-4 (IL-4) and interleukin-6 (IL-6), were measured. Moreover, liver histological examination, antioxidant activity and the concentration of nitric oxide (NO) in liver were assessed. To further understand the mechanism of the protective effect, we evaluated the mRNA expression of hepatic intercellular adhesion molecule-1 (ICAM-1), interferon-inducible chemokine 10 (CXCL10), inducible nitric oxide synthase (iNOS) and B-cell lymphoma-2 (Bcl-2) in liver tissues. We hypothesized that SPE has potential therapeutic effects against immune-mediated hepatitis.

## 2. Results and Discussion

### 2.1. Composition Analysis of SPE

As shown in Table 1, the composition analysis revealed that protein was the main component (84.95%) of SPE. Glutamic acid, aspartic acid, lysine and leucine were the major amino acids contained in SPE, and the content of eight essential amino acids was 31.20 g/100 g of SPE, which constituted 39.48% of all amino acids (Table 2). 

**Figure 1:**
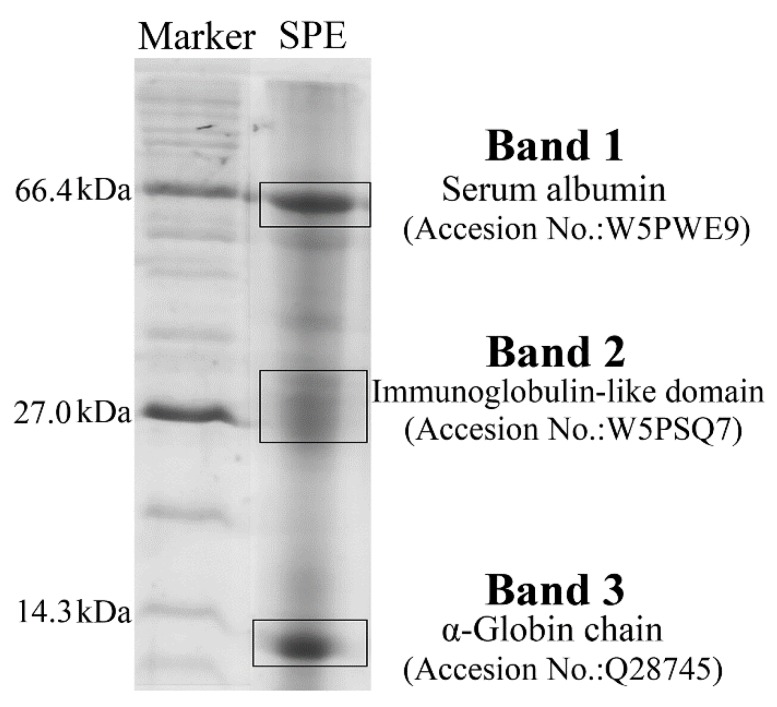
SDS-PAGE profiles and identification of proteins by mass spectrometry in sheep placental extract (SPE). Accession No. is the corresponding numbers of the proteins in UniProt database.

SDS-PAGE and mass spectrometry were performed to determine the main proteins and their molecular weight distribution. There were three main electrophoresis bands in the SDS-PAGE profiles of SPE, with molecular weight around 66.4 kDa, 27.0 kDa and 14.3 kDa (Figure 1). Mass spectrometry analysis of these three prominent bands showed the main proteins around 66.4 kDa were serum albumins, while the proteins around 27.0 kDa were immunoglobulin-like domains. The proteins around 14.3 kDa mainly belonged to the globulin family. The albumins and the immunoglobulin-like domains play important roles in the immune system and inflammation regulation [10]. Apart from the proteins, peptides and other bioactive compounds may also contribute to the bioactivity of placenta extract. Some previous studies have indicated peptides from goat placenta extract and human placenta extract had numerous bioactivities, such as antioxidant and anti-inflammatory activities [11,12,13]. Moreover, some cytokines and growth factors in placenta extract were also found to be essential for tissue regeneration [14,15].

### 2.2. Serum Biochemical Markers 

**Figure 2:**
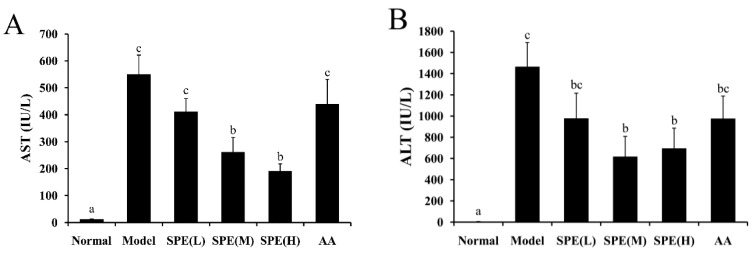
The effects of sheep placental extract (SPE) on serum biochemical markers 8 h after injection of Con A. The activities of alanine transaminase (AST) and aspartate transaminase (ALT) are presented in (**A**,**B**), respectively. Values are represented as means ± SE (*n* = 11). a, b, c and bc in the figures show statistically significant differences (*p* < 0.05) among groups. The experimental groups pretreated with low dose SPE (5 mg/kg), medium dose SPE (10 mg/kg), high dose SPE (50 mg/kg) or the mixture of 18 amino acids equal to 50 mg/kg SPE group are labelled as SPE (L), SPE (M), SPE (H) and AA, respectively.

The Con A-induced liver injury model closely mimics the pathogenic mechanisms in human viral and autoimmune hepatitis. Many indicators, such as pro-inflammatory cytokines, precede the changes in aminotransaminase levels [16]. The serum level of cytoplasmic enzymes such as AST and ALT, which are leaked from damaged liver cells into the blood, is one of the important indicators for hepatic damage. Figure 2 shows the activity levels of AST and ALT in serum. Compared with the normal group, the transaminase levels in the Con A-induced model group increased significantly. The high dose (50 mg/kg) SPE pretreatment significantly reduced the activity levels of AST and ALT by 65.39% and 52.52%, respectively, compared with the model group (*p* < 0.05). Administration of the amino acids mixture produced no significant decrease compared with the Con A-induced model group (*p* > 0.05). The results indicated that pretreatment with SPE ameliorated the severity of hepatic injury caused by Con A, and the effect was mainly attributed to proteins and peptides rather than individual amino acids.

### 2.3. Histological Examination of the Liver

**Figure 3:**
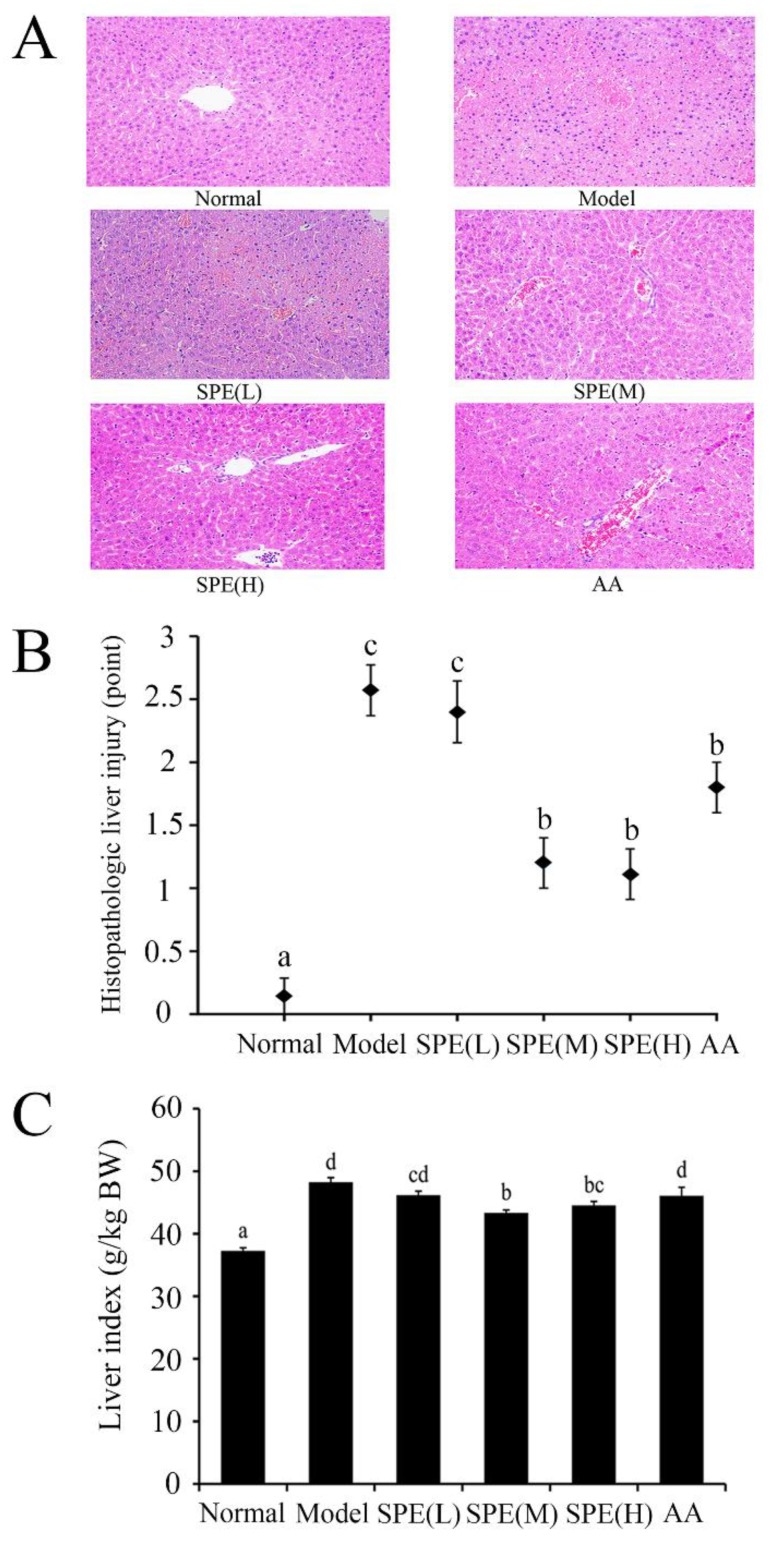
Haematoxylin and eosin (H&E) staining of liver tissue, histological analysis and the liver index in different groups. H&E staining with magnification ×200, histological analysis and the liver index are shown in (**A**–**C**), respectively. Histopathology scores, including hepatocyte swelling, degeneration and inflammatory infiltration, are graded on a three-point severity scale: 0, none; 1, mild; 2, moderate; 3, severe. The liver index is shown as the ratio between the liver weight and body weight (g/kg). Values are represented as means ± SE (*n* = 11). a, b, c, d, bc and cd in the figures indicate statistically significant differences (*p* < 0.05) among groups. The experimental groups pretreated with low dose sheep placental extract (SPE) (5 mg/kg), medium dose SPE (10 mg/kg), high dose SPE (50 mg/kg) or the mixture of 18 amino acids equal to the 50 mg/kg SPE group are labelled as SPE (L), SPE (M), SPE (H) and AA, respectively.

Histological analysis could be used to reveal the extent of hepatocyte swelling, degeneration and inflammatory infiltration in liver. The degree of liver edema was indicated by the liver index. Figure 3 shows representative images of liver H&E staining, histological analyses and liver index in the different groups. When mice were administered with 15 mg/kg Con A for 8 h, the model group suffered extensive liver damages (around grade 2.5) and pronounced lesions were observed in the livers. Pretreatment with the medium dose (10 mg/kg) or the high dose (50 mg/kg) of SPE for 3 d before Con A injection significantly alleviated the injury shown by liver histopathology and efficiently decreased the accompanying liver index (*p* < 0.05). Administration of amino acids equivalent to 50 mg/kg SPE had no significant effect on the liver index (*p* > 0.05). Histological examination of liver tissues further confirmed the serum biochemical findings, indicating that SPE had significant hepatoprotective effects in the Con A-induced immunological liver injury model.

### 2.4. Superoxide Dismutase (SOD) Activity, Malondialdehyde (MDA) Content and NO Content in Liver Tissues

**Figure 4:**
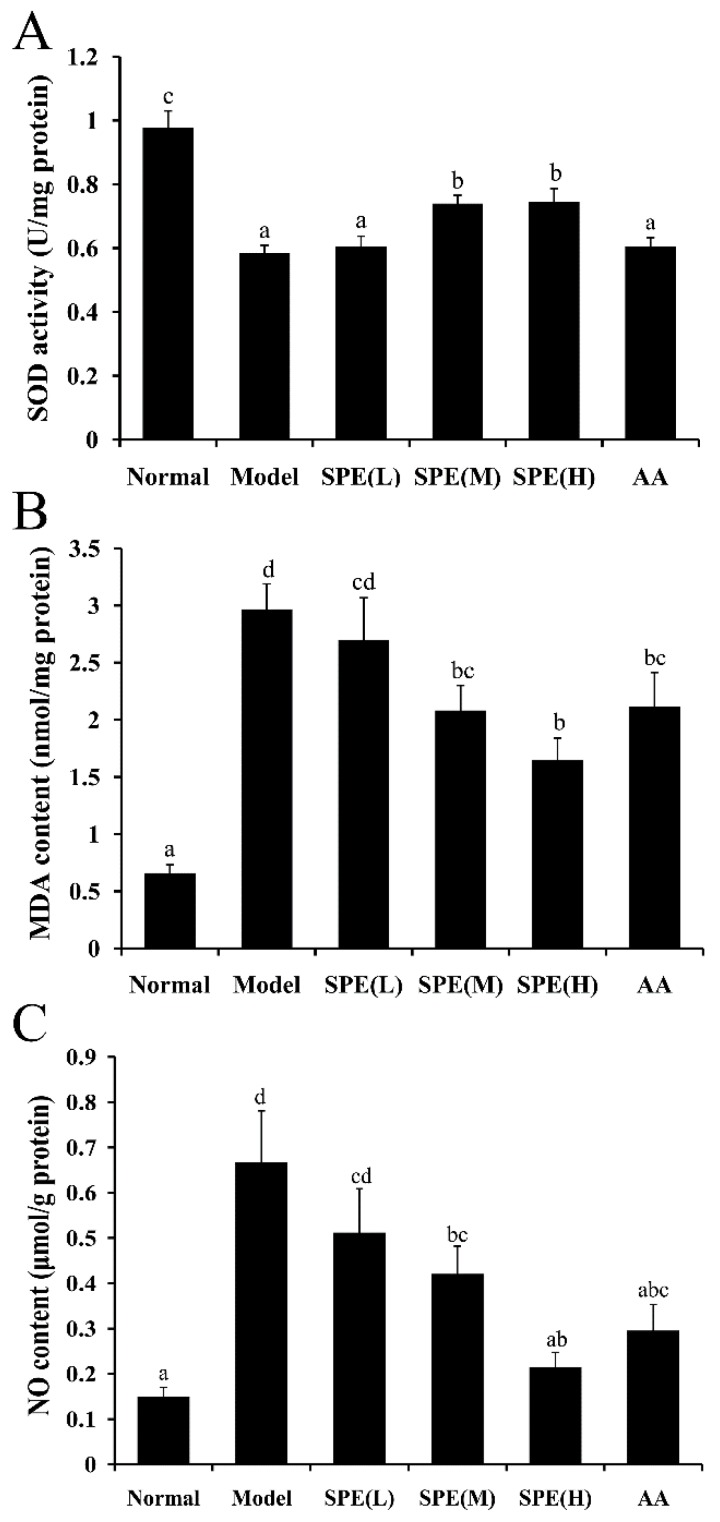
The effects of sheep placental extract (SPE) on the superoxide dismutase (SOD) activity, the malondialdehyde (MDA) content and nitric oxide (NO) content after 8 h of administration of Con A. The activity of SOD, the content of MDA and the content of NO in the liver tissues of Con A-induced mice are shown in (**A**–**C**), respectively. Values are represented as means ± SE (*n* = 11). a, b, c, d, ab, bc, cd and abc in the figures indicate statistically significant differences (*p* < 0.05) among groups. The experimental groups pretreated with low dose SPE (5 mg/kg), medium dose SPE (10 mg/kg), high dose SPE (50 mg/kg) or the mixture of 18 amino acids equal to the 50 mg/kg SPE group are labelled as SPE (L), SPE (M), SPE (H) and AA, respectively.

The capacity of hepatic cells to resist oxidative stress is directly correlated with their function. SOD activity is an important antioxidant parameter in evaluating liver tissue activity. MDA, a major degradation product of lipid hydroperoxides, is another indicator used to assess the extent of lipid peroxidation in liver [17]. Significant hepatocyte oxidative stress (*p* < 0.05) was observed after 15 mg/kg Con A administration, which is shown by decreased SOD activity and increased MDA content in Figure 4A,B. Compared with the Con A-induced model group, pretreatment with high dose (50 mg/kg) SPE increased SOD activity by 27.36% and decreased the MDA content by 44.37%, and the responses were dose-dependent. The results possibly suggested that the protection exerted by SPE is due to its radical scavenging activity. Furthermore, administration of amino acids equivalent to 50 mg/kg SPE did not significantly affect SOD activity but significantly decreased MDA content in liver tissues compared with the Con A-induced model group. This result indicated amino acids prevented hepatocellular lipid peroxidation but had no significant effect on the generation of endogenous antioxidant enzymes. Moreover, the anti-inflammatory pathway Keap1-HO1-CO axis was reported to play a protective role in the development of Con A-induced hepatitis [18]. This may also contribute to the protective effect of SPE, which was consistent with the fact that SPE augments anti-oxidative responses.

NO, a hepatotoxic small molecule produced in Con A-induced models, causes cascade amplification of liver damage [16]. The hepatic NO levels in different treatment groups are shown in Figure 4C. Compared with normal mice, the NO content in the Con A-induced model group increased 400%. The medium dose (10 mg/kg) and high dose (50 mg/kg) SPE pretreatments significantly decreased the content of NO in liver tissues (*p* < 0.05). 

NO may further facilitate tissue injury through several pathways, including mitochondrial respiration inhibition, DNA synthesis disruption and free radicals’ generation. Among these mechanisms, free radicals induced oxidative stress is crucial in liver injury [16]. Excess NO can produce highly reactive peroxynitrite anions, the inhibitory effect of SPE on the NO content suggesting SPE may protect liver cells from injuring through radical scavenging activity.

### 2.5. The Cytokine Content in Serum

**Figure 5:**
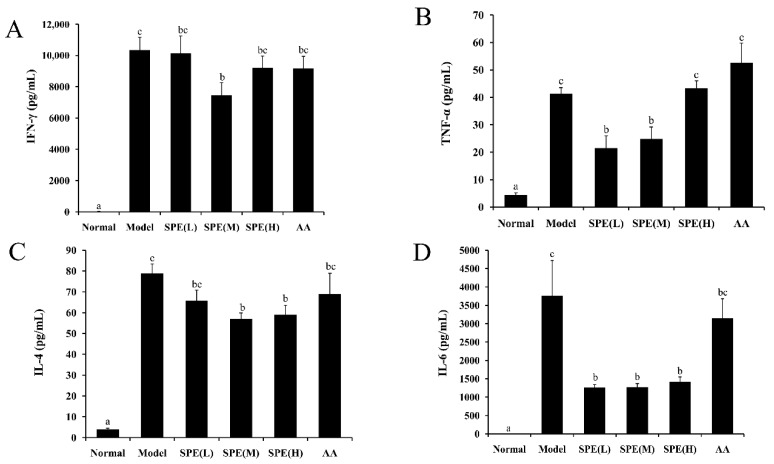
The effects of sheep placental extract (SPE) on the concentration of IFN-γ (**A**), TNF-α (**B**), IL-4 (**C**) and IL-6 (**D**) in serum. Values are represented as means ± SE (*n* = 11). a, b, c and bc in the figures indicate statistically significant differences (*p* < 0.05) among groups. The experimental groups pretreated with low dose SPE (5 mg/kg), medium dose SPE (10 mg/kg), high dose SPE (50 mg/kg) or the mixture of 18 amino acids equal to the 50 mg/kg SPE group are labelled as SPE (L), SPE (M), SPE (H) and AA, respectively.

Inflammatory responses are related with the progression of liver fibrosis [19], and Con-A induced liver injury is characterized by inflammation and immune disorder. Upon Con A stimulation, T cells release a variety of cytokines [20]. Both type 1, type 2 cytokines (with exception of IL-10 that is protective) and Th17 cytokines plays pathogenetic role in Con A-induced liver injury and that IL-4 has actually a pathogenetic effects [21,22,23,24]. Among them, IFN-γ, TNF-α, IL-4 and IL-6 play important roles in the pathogenesis of this model [25]. Figure 5 shows the concentrations of IFN-γ, TNF-α, IL-4 and IL-6 in the serum of different groups. 

Compared with that in the normal group, the cytokine content in the Con A-induced model group was significantly elevated (*p* < 0.05). The medium dose (10 mg/kg) SPE pretreatment significantly decreased the IFN-γ and TNF-α content (*p* < 0.05) compared with the Con A-induced model control. It is still unclear that SPE failed to depress IFN-γ and TNF-α in high dose, and further research is needed. Besides, in present study, the increase of TNF-α was observed after 8 h Con A injection, which was in accordance with some previous publications [26,27]. While, the other researchers found the level of TNF-α peaked 2 h after Con A injection rather than several hours later [28]. The reason for this discrepancy might be attributed to the different stains and environment factors [26]. In addition, the levels of IL-4 and IL-6 in serum also significantly decreased (*p* < 0.05) in the groups pretreated with SPE. The mixture of amino acids produced no significant changes, consistent with the results of serum biochemical markers (ALT and AST) and the liver index measurements. These data indicated the protective role of SPE in Con A-induced hepatitis was correlated with the production of pro-inflammatory cytokines, and that the potential mechanism underlining this beneficial effect involved inhibition of the inflammatory response. Combining the results from some previous studies, it was supposed that a possible mode of SPE action may rely on inhibition of cytokines involved in Con A-hepatitis such as MIF and IL-18 [29,30], or stimulated production of cytokines that are protective in the model such as IL-10 [31].

### 2.6. The Expression of mRNA in Liver Tissues

**Figure 6:**
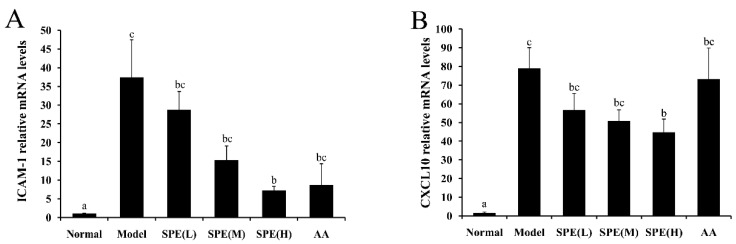

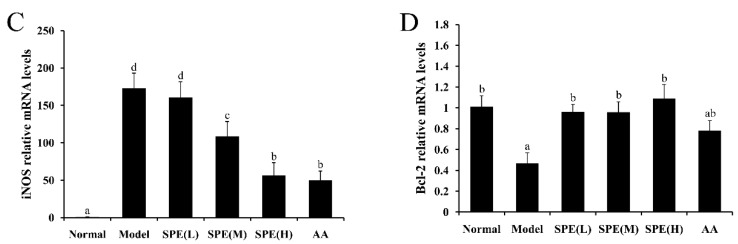
The effects of sheep placental extract (SPE) on the mRNA expression of ICAM-1 (**A**), CXCL10 (**B**), iNOS (**C**) and Bcl-2 (**D**) in liver tissues. The mRNA expression was normalised with the expression of β-actin in each sample. Values are represented as means ± SE (n = 11). a, b, c, d, ab and bc in the figures indicate statistically significant differences (*p* < 0.05) among groups. The experimental groups pretreated with low dose SPE (5 mg/kg), medium dose SPE (10 mg/kg), high dose SPE (50 mg/kg) or the mixture of 18 amino acids equal to the 50 mg/kg SPE group are labelled as SPE (L), SPE (M), SPE (H) and AA, respectively.

The secretion of adhesion molecules is a hallmark in the pro-inflammatory state of leukocytes, and chemokines direct the migration of circulating leukocytes to sites of inflammation or injury [32]. Compared with the Con A-induced model group, pretreatment with SPE showed lower mRNA expression of ICAM-1 and CXCL10 in a concentration-dependent manner, and significant decrease was observed in the high dose SPE treatment group (Figure 6A,B) (*p* < 0.05), which indicated the beneficial effects of SPE may be related to the regulation of T cell distribution [33].

Expression of iNOS is strongly upregulated under pathological conditions [16]. It was reported that pro-inflammatory cytokines induce the expression of iNOS in hepatocytes, and excessive NO derived from iNOS contributes to the development of Con A-induced liver injury [34]. Figure 6C shows the effects of pretreatment with SPE on the mRNA expression of iNOS. The Con A-induced mRNA expression of iNOS was significantly reduced by the medium/high dose of SPE and mixture of amino acids (*p* < 0.05), which explained the NO content results.

The expression of Bcl-2, an essential anti-apoptotic protein, plays a key role in the regulation of cell proliferation and apoptosis, as well as inflammatory injury [35]. The injection of Con A could lead to the increase of hepatocyte apoptosis, which is a crucial mechanism leading to hepatic dysfunction [36]. The mRNA expression level of Bcl-2 was measured to investigate the effect of SPE treatment on the liver cells apoptosis. It showed that pretreated with SPE, rather than the mixture of amino acids, significantly promoted the mRNA expression of Bcl-2 (Figure 6D), which further suggested the protective effect of SPE on Con A-induced liver cell apoptosis.

## 3. Materials and Methods 

### 3.1. Materials

Fresh sheep placentas were collected from Caoyuanxinhe Food Co., Ltd. in Bayan Nur City, Inner Mongolia Province, China. Con A and 18 amino acids (aspartic acid, threonine, serine, glutamic acid, proline, glycine, alanine, valine, methionine, isoleucine, leucine, tyrosine, phenylalanine, histidine, lysine, arginine, cystine and tryptophan) were purchased from Sigma-Aldrich Corporation (St. Louis, MO, USA).

### 3.2. Extraction of Sheep Placenta

The extraction of sheep placenta was based on the method described in a previous study [37], in which the endotoxin contamination was rarely seen. The amnion and cord of fresh sheep placenta were discarded and the remaining tissue was completely washed in distilled water to remove all blood debris. The placentas were cut into small pieces (<1 cm^2^), suspended in phosphate-buffered saline (PBS, 0.01 M, pH 7.4) at a ratio of 1:2.5 (*w*/*v*) and homogenized for 5 min. To prepare soluble fractions, tissue homogenates were centrifuged at 5000 rpm for 20 min at 4 °C. The supernatant was dialyzed against distilled water for 24 h at 4 °C, with several water changes to remove excessive salt. The extracts were then lyophilised and the dried SPE materials were stored at −20 °C before experiment.

### 3.3. Composition Analysis of SPE

The contents of total water, protein, fat, ash and carbohydrate in SPE were determined according to the corresponding Chinese National Standards (GB/T 5009.3-2010, GB/T 5009.5-2010, GB/T 5009.6-2003, GB/T 5009.4-2010 and GB/T 9695.31-2008). Amino acids were analysed by an L-8900 automatic amino acid analyser (Hitachi, Tokyo, Japan). The proteins in SPE were also analysed by SDS–PAGE (12.5%) and the main electrophoresis bands were identified by Triple TOF 5600-plus mass spectrometry (SCIEX, Framingham, MA, USA). 

### 3.4. Animals and Experiment Design 

Female, 6-wk old BALB/c mice were purchased from Beijing Vital River Company (Beijing, China). Mice were housed in a conventional temperature and humidity-controlled room and they were fed with a standard rodent diet (Beijing HFK Bioscience Co., Ltd., Beijing, China) and water. They were acclimated for 1 wk prior to experimentation. The mice were randomly divided into 6 groups (*n* = 11 per group): a normal group (vehicle), the model group (vehicle plus 15 mg/kg body weight of Con A), SPE groups (5 mg/kg, 10 mg/kg or 50 mg/kg body weight of SPE plus 15 mg/kg body weight of Con A), and the AA group (18 amino acids mixtures plus 15 mg/kg body weight of Con A). The concentrations of 18 amino acids referred to the amino acids composition measured in 50 mg/kg SPE and the AA group was set to explore if amino acids were responsible for the hepatoprotective effects of SPE. SPE, Con A and the 18 amino acids were dissolved in pyrogen-free PBS. SPE or the mixture of amino acids was administered orally once per day for 3 days, and Con A was administered by intravenous injection 1 h after the final administration of SPE or the mixture of amino acids. The mice were sacrificed 8 h after the administration of Con A.

### 3.5. Measurement of Biochemical Markers in Serum 

Blood was collected 8 h after Con A administration and the serum was separated by centrifugation at 4000× *g* for 15 min after a 2 h incubation at room temperature. The activities of serum ALT and AST were measured using assay kits (Nanjing Jiancheng Bioengineering Institute, Nanjing, China). The assays were performed following the manufacturer’s instructions.

### 3.6. Liver index and Histopathological Analysis

The body weight of each mouse was determined before the experiment and the liver index was calculated as the liver weight (g) divided by the body weight of the mice (kg). For histopathological analyses, the left lateral liver lobes were obtained 8 h after Con A administration and fixed in 10% (*v*/*v*) buffered formalin for 48 h. The formalin fixed tissue was embedded in paraffin, dehydrated with 80% ethanol, and 5 µm sections were stained with haematoxylin and eosin (H&E). Three visual fields were randomly selected for each section (×200). Histopathology was assessed by two experienced pathologists. Histopathological changes, including pericentral, lobular inflammation and centrilobular necrosis, were graded on a severity scale from 0 to 3 (0, no lesion; 1, mild; 2, moderate; 3, severe) [17].

### 3.7. Measurements of SOD, MDA and NO in Liver Tissue

The liver tissue was harvested 8 h after Con A administration and homogenised in pyrogen-free saline, on ice, and then the SOD activity, MDA and NO content in liver homogenate were measured. The assay kits for SOD activity (Beyotime Institute of Biotechnology, Beijing, China), MDA and NO content (Nanjing Jiancheng Bioengineering Institute, Nanjing, China) were used according to the manufacturer’s instructions. The content of protein was determined using the bicinchoninic acid protein assay [38].

### 3.8. Serum Cytokine Measurement

Serum was obtained 8 h after Con A administration and the concentrations of TNF-α, IFN-γ, IL-4 and IL-6 were measured using enzyme-linked immunosorbent assay (ELISA) kits (Thermo Fisher Scientific, Waltham, MA, USA), according to the manufacturer’s instructions.

### 3.9. Real-time PCR Analysis

Liver samples were collected 8 h after Con A administration and homogenised in liquid nitrogen. Total RNA was isolated by TRNzol reagent (Tiangen Biotech Company, Beijing, China) according to the protocol provided by the manufacture, then cDNA was synthesized from 4 µL of total RNA using 5× All-In-One RT Mastermix (Applied Biological Materials Incorporated, Vancouver, Canada). Real-time PCR was performed using a SYBR^®^ Prime ScriPTM reagent kit (TaKaRa, Dalian, China). The primers used were as follows: β-actin (forward, F) 5′-CTGGCACCACACCTTCTAC-3′ and (reverse, R) 5′-GGGCACAGTGTGGGTGAC-3′; ICAM-1 (F) 5′-CCATCACCGTGTATTCGTTT CC-3′ and (R) 5′-CTGGCGGCTCAGTATCTCCTC-3′; CXCL10 (F) 5′-TGAAATCACCCTGCGAGCCT AT-3′ and (R) 5′-GCACCTTGGAAGCCCTACAG-3′; iNOS (F) 5′-GAGCCACAGTCCTCTTTGCTA-3′ and (R) 5′-TGTCACCACCAGCAGTAGTTG-3′; Bcl-2 (F) 5′-CCATCCACTTGTGGCCCAGGTATGC-3′ and (R) 5′-CGCCGGGCTGGGGATGACTTCT-3′ [33,39,40]. The cycles for PCR were as follows: 95 °C for 30 s, then 40 cycles of 95 °C for 10 s, 55 °C for 10 s and 72 °C for 30 s. β-Actin was used as an internal control and target gene expression levels were calculated with the 2^−ΔΔCT^ method [41].

### 3.10. Statistical Analysis

SPSS version 17.0 (SPSS Inc., Chicago, IL, USA) was used for data analysis. Differences among groups were determined by one-way analysis of variance (ANOVA) followed by Duncan’s test. A *p*-value < 0.05 was considered significant.

## 4. Conclusions

In this study, we found that pretreatment of SPE significantly ameliorated the liver injury in a dose-dependent manner in mice induced by Con A. We observed a heptoprotective effect of SPE treatment through decreased level of AST/ALT in blood serum, lower histopathologic liver injury points and smaller liver index. Moreover, the pretreatment with SPE could increase SOD activity, decrease the contents of MDA and NO in the liver tissues. The protective effect of SPE on Con A-induced hepatitis may be functioned through increased radical scavenging activity, decreased cytokine secretion and lower mRNA expression of adhesion molecules as well as chemokines by SPE pretreatment. The increased anti-apoptotic activity brought by SPE also contributed to such protective behaviour towards liver tissues. The findings demonstrate the potent activity of SPE against Con A-induced liver injury. Therefore, SPE may be a therapeutic candidate for treatment of immune-mediated hepatitis.

## Figures and Tables

**Table 1:** Composition of sheep placental extract. ^1^

Category	Content (g/100 g)
Water	4.07 ± 0.31
Crude protein	84.95 ± 0.81
Total fat	2.08 ± 0.04
Ash	5.53 ± 0.09
Total carbohydrate	2.06 ± 0.03

^1^ The results are averages of three independent experiments and the data are expressed as mean ± SD.

**Table 2:** Amino acids content in sheep placental extract.

Amino Acids	Content (g/100 g)	Amino Acids	Content (g/100 g)
Aspartic acid	8.00	Isoleucine	2.74
Threonine	4.01	Leucine	6.88
Serine	4.07	Tyrosine	2.72
Glutamic acid	12.51	Phenylalanine	3.97
Proline	3.78	Histidine	2.32
Glycine	3.78	Lysine	6.90
Alanine	4.17	Arginine	4.79
Valine	4.14	Cystine	1.69
Methionine	1.56	Tryptophan	1.00
